# The once and future gene therapy

**DOI:** 10.1038/s41467-020-19505-2

**Published:** 2020-11-16

**Authors:** Karen Bulaklak, Charles A. Gersbach

**Affiliations:** 1 Department of Biomedical Engineering, Duke University, Durham, NC USA; 2 Center for Advanced Genomic Technologies, Duke University, Durham, NC USA; 3 Department of Surgery, Duke University, Durham, NC USA; 4 Department of Cell Biology, Duke University, Durham, NC USA

**Keywords:** Gene delivery, Gene therapy

## Abstract

Gene therapy is at an inflection point. Recent successes in genetic medicine have paved the path for a broader second wave of therapies and laid the foundation for next-generation technologies. This comment summarizes recent advances and expectations for the near future.

The past five years have seen a renaissance in the field of gene and cell therapy and the first approved therapies following decades of efforts (Fig. 1). This includes the first oligonucleotide-based therapies (Spinraza, Exondys 51, Vyondys 53), three cell therapies (Kymriah, Yescarta, Tescartus), and two in vivo gene therapies (Luxturna and Zolgensma), with more following close behind. These therapies treat diverse clinical indications and tissue targets, including neuromuscular disease, inherited blindness, and cancer. While these approved therapies are life-changing for the affected patients, they offer even broader impact in what they demonstrate more generally for the field and lay a foundation on which treatments for many other conditions can be developed. For example, the success of in vivo AAV gene transfer to the human retina and central nervous system by Luxturna and Zolgensma for Leber’s congenital amaurosis and spinal muscular atrophy, respectively, has facilitated the development of AAV-based therapies for gene delivery to the liver and skeletal muscle to treat hemophilia^1^ and Duchenne muscular dystrophy^2^, respectively. Similarly, early technology development in ex vivo lentiviral and retroviral gene transfer to T cells that led to adoptive cell immunotherapy has been expanded to modification of hematopoietic stem cells, enabling therapies for inherited disorders such as sickle cell disease and beta thalassemia recently approved in the European Union and currently under review in the United States^3^.

**Fig. 1:**
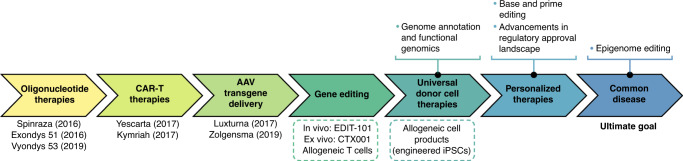
Timeline depicting milestones (in colored arrows) towards gene therapies for common disease. Approved treatments and year of their approval as well as investigational therapies (in dashed boxes) are shown below each milestone. Further exploration of alternative therapeutic approaches and fundamental scientific questions is still needed to accomplish later milestones (shown in bullets).

While the potential for these early gene therapy successes to be extrapolated to other conditions and patient populations is exciting, next-generation technologies are dramatically expanding the impact of these medicines on treating human disease (Fig. 2). For example, a primary obstacle to broader application continues to be the immune response to gene delivery vectors and products of foreign transgenes. Accordingly, control of the human immune system is where some of the most impactful work will occur in the near future. For example, despite the striking success of many AAV-based gene therapies, as many as 50% of patients are currently excluded from treatment due to pre-existing immunity to the viral capsids^4^. Recent advances and efforts currently in clinical trials have led to technological advances to circumvent this immune obstacle, such as engineering of modified AAV capsids that evade pre-existing neutralizing antibodies^5,6^ and methods for temporary clearing of antibodies from circulation^7^. Immunosuppression regimens may also provide a means for both circumventing pre-existing immunity and avoiding adaptive immunity to the vector, which may enable subsequent re-dosing if necessary^8,9^.

**Fig. 2:**
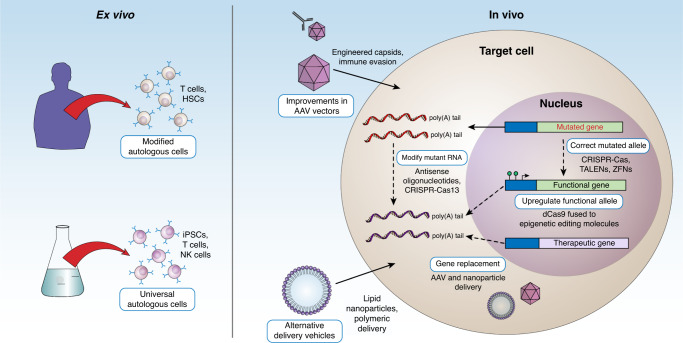
Schematic of ex vivo and in vivo strategies (shown in blue boxes) for treating genetic diseases. For ex vivo approaches (left panel), autologous cells can be isolated directly from the patient and genetically modified to elicit a therapeutic effect, while allogeneic cells can be produced and readily available “off the shelf.” In vivo strategies require targeting of specific cells in order to overexpress a therapeutic gene or correct pathological mechanisms to allow functional gene expression (dashed arrows).

Additionally, the engineering and profiling of non-viral nanoparticles for gene delivery has undergone remarkable advances and increases in throughput^10^. These technologies are likely to have a tremendous impact on gene therapy in the future by building on the clinical success of nanoparticle-delivery of siRNAs and the first approval of an siRNA-based drug, Onpattro for the treatment of hereditary ATTR amyloidosis, in 2018^11^. One possible advantage of nanoparticles is the potential to circumvent detection by the immune system that limits viral delivery. Additionally, chemically defined nanoparticle formulations present unique opportunities for functionalization and tissue targeting that may ultimately be critical to success of in vivo gene transfer outside of the retina and liver.

Beyond the first generation of gene therapies that have focused on delivery of transgenes, gene editing technologies are enabling an entirely new modality for treatments based on precise modification of human genome sequences. While gene editing therapies first entered clinical trials in 2010 as an approach to prevent HIV infection of T cells^12^, the first example of disease-modifying efficacy was demonstrated only in the past year in clinical trials of CRISPR-based gene editing for sickle cell disease and beta-thalassemia (CTX001)^13^. This pioneering success, combined with a promising safety record thus far for gene-edited T cells and HSCs in human trials^13–15^, has set the stage for highly anticipated results from ongoing and imminent clinical trials of in vivo genome editing, including a current trial of AAV-based gene editing in the retina (EDIT-101)^16^ and a planned trial for non-viral nanoparticle based delivery of CRISPR to the liver (NTLA-2001)^13,17^. Nevertheless, expanding to target tissues outside of the retina and liver comes with many challenges. To this point, the National Institutes of Health has announced a commitment of $190 million over six years to support a Somatic Cell Genome Editing Consortium that will directly address the challenges of delivery, safety, and modeling of systems to advance in vivo genome editing to broadly address human health disorders across diverse tissue types and disease conditions. Assuredly, the products of this Consortium will significantly accelerate the progression of gene editing therapies over the next ten years and beyond.

Current gene editing technologies use nuclease-based systems to cut DNA strands and stimulate DNA repair pathways to introduce desired sequence changes. While these technologies are only currently beginning to be tested clinically, multiple waves of next-generation editing technologies are lined up at the heels of these efforts to improve specificity, accuracy, efficiency, and applicability to different classes of disease^18,19^. For example, the inventions of base editing and prime editing have enabled the precise alteration of genomic sequences in the absence of DNA breaks and without the reliance on the activity of endogenous DNA repair pathways^18^. RNA-targeted editing technologies allow for transient and reversible modification of gene expression without necessitating permanent changes to genome sequences, potentially leading to greater efficiency and safety^19^. Finally, epigenome editing technologies have the advantage of tunability, reversibility, and the potential for sustained outcomes after transient editor activity that are heritable through cell division^20^. In parallel to these advanced editing modalities, the roster of possible DNA-targeting systems continues to expand, particularly with the exponentially increasing diversity of CRISPR-Cas systems derived from engineered variants, various bacterial species, and distinct classes of CRISPR targeting mechanisms^19^. The rapid pace of technological innovation in these editing fields is certain to both transform how we currently think about gene therapies but also dramatically broaden the scope of human disease to which these approaches can be applied.

Another area of innovation that will dramatically impact the gene therapy field in the near future is functional genomics and our understanding of the regulation of the human genome. For example, the function of ~6,000 of the ~20,000 human genes is currently unknown^21^. In parallel to enabling gene editing therapies, CRISPR technologies are also facilitating the functional dissection of these gene sequences^22^. Moreover, scientific studies and therapeutic interventions have traditionally focused almost exclusively on genes, even though 98% of our genome consists of non-coding DNA that harbors epigenetic regulators responsible for >90% of susceptibility for common disease^23^. In fact, the first example of therapeutic efficacy of a CRISPR gene editing approach (CTX001) involves the editing of a distal gene regulatory element to alter gene expression, rather than the editing of the underlying genetic mutation, as a strategy to compensate for lost beta globin function in hemoglobinopathies^24^. While efforts such as the NIH ENCODE Consortium have mapped more than two million of these gene regulatory elements across hundreds of human cell types and tissue samples, the function of very few of these sites is known^25^. Annotating this dark matter of the genome will lead to whole new areas of disease biology and classes of therapeutic targets that will enable attacking human disease from an entirely new angle with gene therapy, gene editing, and other modalities.

Remarkably, the rate of technological innovation of gene and cell therapy is significantly outpacing the ability to safely and expeditiously move promising candidates forward in order to benefit patients. Current regulatory models that require large numbers of patients to establish safety and efficacy are not applicable to curative technologies that address a mutation that is found in a single patient or very few patients. One promising strategy is to create a single composition that can treat a larger patient population. Universal cell therapies, which are generated by applying gene editing to engineer “immune stealth” allogeneic donor cells that evade the detection of the host immune system, can be used in both regenerative medicine and adoptive cell immunotherapy^26^. Several clinical trials are currently underway to investigate therapies of this design^13^, and the readout of results from those trials in the near future will significantly shape the future of gene and cell therapy. Despite the promise of this approach, it does not address correction of genetic mutations in vivo and does not leverage the tremendous opportunity and increasing efforts in developing transformative technologies such as base editing and prime editing, which have the potential to correct individual private mutations. In a similar vein, the recent report of an oligonucleotide-based therapy targeted to a private genetic mutation and successful treatment of a human Batten disease patient provides both a potential blueprint and motivation for these efforts^27^. Consequently, one of the greatest changes in the field of cell and gene therapy in the near future will be in the area of regulatory sciences and accommodating the unique challenges posed by these innovative technologies as we move toward personalized therapies.

Gene therapy is arguably the most exciting area of biotechnology at this moment - both due to recent progress and because of the possibilities on the horizon. Unprecedented levels of control over nucleic acid delivery, modulation of the immune system, and precise manipulation of the human genome – technologies not imaginable ten years ago – will certainly unlock new areas of medicine over the next ten years. At the same time, this nascent glimpse of a new world of technical capabilities has inspired whole new areas of research, such as synthetic biology, cell reprogramming, and high-throughput functional genomics, which will undoubtedly continue to reshape the face of biomedical research.
